# Diagnostic and Prognostic Utility of Cell-Surface Vimentin Positive Circulating Tumor Cells in Breast Cancer Using an Automated Negative Selection Platform

**DOI:** 10.3390/diseases14040130

**Published:** 2026-04-03

**Authors:** Ming-Hsin Yeh, Mei-Chun Lin, Hui-Ju Tsai, Yi-Chou Liu, Tzu-Min Wang, Wei-Shan Hung, Chih-Peng Lin, Ching-Hsing Liang, Chih-Jen Tseng

**Affiliations:** 1 Division of Breast Surgery, Department of Surgery, Chung Shan Medical University Hospital, Taichung City 40201, Taiwan; breast5321@gmail.com (M.-H.Y.); meichun1202@gmail.com (M.-C.L.); 2 Institute of Medicine, Chung Shan Medical University, Taichung City 40201, Taiwan; 3 Good Future Biomedical Technology Corp., Taoyuan City 33377, Taiwan; amber@gfmed.cc (H.-J.T.); daniel.liu@gfmed.cc (Y.-C.L.); carol@gfmed.cc (T.-M.W.); sarah@gfmed.cc (W.-S.H.); lin.darren@gfmed.cc (C.-P.L.); douglas@gfmed.cc (C.-H.L.); 4 Obstetrics and Gynecology, Department of Surgery, Chung Shan Medical University Hospital, Taichung City 40201, Taiwan

**Keywords:** breast cancer, metastatic risk, recurrence, circulating tumor cells, epithelial cell adhesion molecule (EpCAM), cell-surface Vimentin (CSV)

## Abstract

**Background/Objectives:** Breast cancer (BC) is the most commonly diagnosed cancer in women, and metastasis is the leading cause of BC-related death. Circulating tumor cells (CTCs) are a prerequisite for metastasis. This study examined the diagnostic and prognostic value of CTCs for assessing metastatic risk and recurrence in BC. **Methods:** The Chiline CATCH^®^ Circulating Target Cell Enrichment System, an automated negative selection platform, was used to enrich and enumerate CTCs from the peripheral blood of patients with BC. Epithelial cell adhesion molecule (EpCAM) and cell-surface Vimentin (CSV) were used as markers for CTC identification. **Results:** CSV^+^ CTC counts, but not EpCAM^+^ CTC counts, were increased in patients with BC at higher metastatic risk. A cut-off of >4.5 CSV^+^-CTCs/2 mL blood yielded a sensitivity of 0.56 and specificity of 0.92 for identifying patients at high metastatic risk. CSV^+^-CTCs outperformed conventional serum tumor markers, including cancer antigen 15-3 (CA 15-3), cancer antigen 125 (CA 125), and carcinoembryonic antigen (CEA), in identifying patients with high metastatic risk, and their combined use further improved risk stratification. An elevated CSV^+^-CTC count (≥5 cells/2 mL blood) was significantly associated with worse progression-free survival in patients with BC. **Conclusions:** These findings suggest that CSV^+^-CTCs may serve as a biomarker for metastatic risk stratification and recurrence monitoring in BC when measured using an automated negative selection platform.

## 1. Introduction

Breast cancer (BC) is the most commonly diagnosed cancer and the leading cause of cancer death in women [1]. Surgical resection and adjuvant systemic therapy are standard treatments for patients with early-stage BC [2,3]. Although early detection and treatment can reduce mortality, some patients still develop recurrence or metastasis during long-term follow-up [4,5]. Metastasis is the primary cause of death in BC and results from the dissemination of cancer cells from the primary tumor to distant organs.

Regular mammography is recommended during follow-up after a diagnosis of BC, but its sensitivity is reduced by approximately 40% in women with dense breast tissue [6,7,8]. Breast ultrasound also plays an important role in diagnosis because it is relatively inexpensive, does not involve ionizing radiation, and allows real-time imaging [9]. However, interpretation of breast ultrasound can be challenging because image appearance varies with operator expertise, equipment, and patient anatomy [10]. This variability may reduce diagnostic accuracy and increase false-positive findings [11,12], leading to unnecessary biopsies, anxiety, and additional healthcare costs. In addition, serum tumor markers such as cancer antigen 15-3 (CA 15-3), cancer antigen 125 (CA 125), and carcinoembryonic antigen (CEA) are non-invasive, readily available, and cost-effective biomarkers for monitoring recurrence and treatment response in BC [13,14,15,16,17]. However, their sensitivity is limited, and marker levels remain within the normal range in a substantial proportion of patients throughout disease progression. Therefore, new biomarkers for detecting systemic micrometastasis are needed.

Circulating tumor cells (CTCs) are cancer cells that detach from primary or metastatic tumors and enter the bloodstream [18]. If they resist shear stress and escape the immune system attack, CTCs can extravasate and establish new metastatic sites. CTCs are therefore considered a prerequisite for cancer metastasis [19]. In addition, CTCs are an important component of liquid biopsy and provide a dynamic and non-invasive approach to cancer diagnostics and management [20,21,22]. Therefore, CTCs are promising candidates for the detection of systemic micrometastasis.

Various approaches have been developed to enrich CTCs based on differences in their biological markers and physical properties [23,24,25]. Cell size, density, and dielectric properties have been used in physical property-based methods for CTC enrichment, but these approaches often yield relatively low recovery rates [26,27]. Biological property-based techniques are mainly divided into positive selection and negative selection. Epithelial markers, such as EpCAM and cytokeratins (CKs; including CK8, CK18, and CK19), are frequently used for the positive selection of epithelial CTCs [28,29,30]. However, epithelial cells can undergo epithelial-mesenchymal transition (EMT), resulting in downregulation of epithelial markers and upregulation of mesenchymal markers such as N-cadherin and Vimentin [31]. Consequently, positive selection techniques that capture only epithelial-positive CTCs may yield false-negative results when tumor cells undergo EMT [24,32]. In negative selection, anti-CD45 antibodies are used to deplete leukocytes and thereby enrich CTCs [33,34]. The advantage of negative selection is that both epithelial-positive CTCs and epithelial-negative CTCs are retained [33]. Epithelial-positive CTCs such as EpCAM^+^-CTCs have been detected in patients with BC, and their applications in early cancer detection, prediction of treatment response, and survival assessment have been reported [35,36,37,38]. However, the role of mesenchymal-positive CTCs such as cell-surface Vimentin-expressing (CSV^+^) CTCs in BC remains incompletely understood, particularly in studies using negative selection-based enrichment strategies. Vimentin has been described in at least two forms: the major intracellular structural form and the functional cell-surface form (CSV), the latter constituting only a minor fraction of total cellular Vimentin. Although intracellular Vimentin has long been recognized as a hallmark of the EMT, the clinical significance of CSV^+^-CTCs in cancer progression has increasingly been recognized over the past decade. Higher CSV^+^-CTC counts have been associated with poorer treatment outcomes and/or worse prognosis in colorectal cancer [39,40], prostate cancer [40,41], gastric cancer [42] and hepatocellular carcinoma [43]. CSV^+^-CTCs have also been reported as a biomarker for risk stratification in head and neck cancer [44] and for predicting distant metastasis in pediatric osteosarcoma and Ewing sarcoma [45]. In this study, we targeted CSV using the highly specific monoclonal antibody clone 84-1, which selectively recognizes the EMT-associated CSV [39]. Unlike its intracellular counterpart, CSV is absent from the surface of normal blood cells, thereby enhancing the specificity of CTC identification and minimizing false-positive counts from leukocytes [46]. Therefore, this study used a negative selection technique to enrich a heterogeneous population of CTCs encompassing both EpCAM^+^-CTCs and CSV^+^-CTCs subtypes and to evaluate their respective roles in the metastatic risk and recurrence of patients with BC.

In this study, the automated negative selection platform, the Chiline CATCH^®^ Circulating Target Cell Enrichment System was used to enrich and enumerate CTCs. The analytical performance of the system, including its stability and reproducibility across independent working units, was first validated. Subsequently, EpCAM^+^-CTCs and CSV^+^-CTCs counts were analyzed to evaluate their associations with metastatic risk and disease recurrence in patients with BC. In parallel, conventional serum tumor markers, including CA 15-3, CA 125, and CEA, were measured and compared with CTC counts to assess their relative diagnostic value for metastatic risk stratification and recurrence monitoring. The implications of these findings are discussed.

## 2. Materials and Methods

### 2.1. Study Subjects

This study was approved by the Institutional Review Board of the Chung Shan Medical University Hospital (IRB No. CS2-22148). All patients provided written informed consent before enrollment. Patients and their clinical data were collected between April 2023 and November 2024. A total of 29 patients with BC who underwent surgery or chemotherapy were enrolled from the Department of Breast and Thyroid Surgery at Chung Shan Medical University Hospital. Patients with BC were stratified into two metastatic-risk groups according to their clinical status at enrollment. The low-risk group (n = 13) included patients with stage 0-II disease or no recurrence for more than five years. The high-risk group (n = 16) included patients with stage III-IV disease; this classification was based on pathological stage at baseline, regardless of whether recurrence had already occurred. Detailed patient characteristics are provided in Supplementary File S1.

### 2.2. Reagents

The Circulating Tumor Cell Enrichment Kit (Epithelial) and the Circulating Tumor Cell Enrichment Kit (Mesenchymal) were purchased from Good Future Biomedical Technology Corp. (Taoyuan, Taiwan). The Elecsys CA 15-3 II assay, Elecsys CA 125 II assay, and Elecsys CEA assay were purchased from Roche Diagnostics Ltd. (Rotkreuz, Switzerland).

### 2.3. Circulating Tumor Cells (CTC) Enrichment and Enumeration

**Figure 1:**
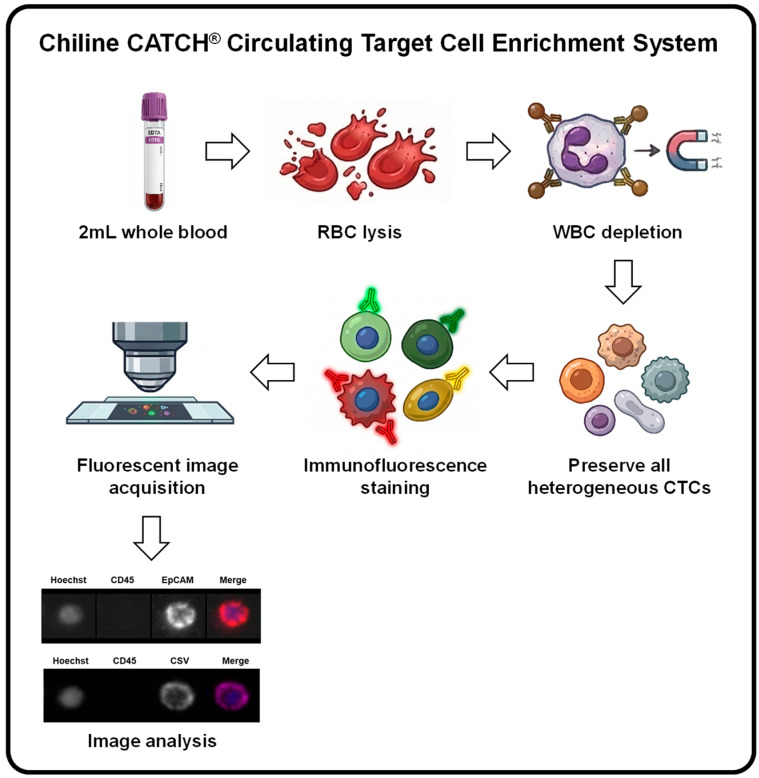
Flowchart of the Chiline CATCH^®^ Circulating Target Cell Enrichment System. Red blood cell (RBC) lysis was performed on 2 mL whole blood samples, and CD45-expressing white blood cells (WBCs) were then removed through immunomagnetic bead-based depletion. The remaining CD45-depleted cells were then subjected to immunofluorescence staining for CTC identification. Immunofluorescent images were captured and analyzed using the integrated microscopy and software in this system. Images were generated using the Gemini 3 Flash model (Google, Mountain View, CA, USA).

CTCs were enriched and enumerated using the automated negative selection platform Chiline CATCH^®^ Circulating Target Cell Enrichment System (Figure 1) (Inventec Appliances Corp., Taoyuan, Taiwan), according to the manufacturer’s instructions (Good Future Biomedical Technology Corp., Taoyuan, Taiwan). All blood samples were obtained via peripheral venous puncture. In brief, red blood cells in 2 mL whole blood samples were lysed, and CD45^+^-leukocytes were depleted using an immunomagnetic bead-based separation method. The remaining CD45-depleted cells were then subjected to immunofluorescence staining for CTC identification. For epithelial CTCs, cells were incubated with Q2 buffer (containing Hoechst 33342, FITC-conjugated anti-CD45, and anti-EpCAM antibodies) for 1 h at room temperature, followed by incubation in the dark for 30 min with R2 buffer (containing Alexa Fluor 594-conjugated secondary antibody). The specificity of the anti-EpCAM antibody is shown in Supplementary Figure S1. For mesenchymal CTCs, cells were incubated for 1 h with S2 buffer (containing Hoechst 33342, FITC-conjugated anti-CD45, and APC-conjugated anti-CSV (clone 84-1) [46] antibodies). After unbound antibodies were removed, immunofluorescent images were captured and analyzed using the integrated microscopy and software of the Chiline CATCH^®^ Circulating Target Cell Enrichment System. EpCAM^+^-CTCs were defined as Hoechst^+^/EpCAM^+^/CD45^−^ cells, and CSV^+^-CTCs were defined as Hoechst^+^/CSV^+^/CD45^−^ cells (Figure 1).

### 2.4. Analysis of CTC Recovery Rate and Leukocyte Depletion by Spiking Test

To evaluate CTC recovery and leukocyte depletion efficiency, a spiking assay was performed using the SKBR3 human breast cancer cell line and the NB4 human acute promyelocytic leukemia cell line. NB4 cells were used as a standardized surrogate for primary WBCs because they constitutively express the CD45 leukocyte common antigen [47], a standard marker of the myeloid lineage. The use of a cell line instead of donor-derived whole blood minimized inter-donor variability and improved experimental reproducibility. In this assay, SKBR3 cells were used as a CTC model and were pre-labeled with Calcein red-orange dye (Thermo Fisher Scientific, Waltham, MA, USA, Cat. No. C34851). A known number of SKBR3 cells was spiked into 1 × 10^7^ NB4 cells in 2 mL reaction buffer. After CTC enrichment and Hoechst 33342 staining, SKBR3 cells were identified by dual positivity for Calcein Red-Orange and Hoechst 33342. Residual NB4 cells were quantified by counting Hoechst 33342-stained nucleated cells to determine depletion efficiency.

In the limit of detection (LoD) assay, cell spiking was not performed using a series of dilutions. To determine the LoD of the Chiline CATCH^®^ Circulating Target Cell Enrichment System for CTCs, SKBR3 cells stained with Calcein red-orange dye were first seeded onto coverslips and observed under a fluorescence microscope. Individual cells were then counted, and the desired number of cells (1 to 5 cells) was carefully transferred into the background NB4 cell suspension for the recovery assay. This approach allowed precise control over the exact number of cells introduced in each experiment. Each condition was tested in independent replicates to ensure reproducibility.

### 2.5. Preparation of Interference Samples for CTC Enrichment and Enumeration

Hemolyzed blood samples were prepared by repeatedly aspirating whole blood through a needle to induce hemolysis. A hemoglobin concentration of 500 mg/dL was defined according to the Hemolysis Reference Palette. Lower levels of hemolysis (50 and 250 mg/dL) were obtained by diluting non-hemolyzed control samples with autologous plasma to achieve the desired hemoglobin concentrations. To prepare lipidemic samples, triglyceride stock (Sigma-Aldrich, St. Louis, MO, USA, Cat. No. 17810) was added to whole-blood samples. Final triglyceride concentrations of 500 and 1000 mg/dL were achieved by directly spiking the stock solution into non-hemolyzed whole blood. To prepare clotting samples, whole blood was collected into BD Vacutainer urine tubes (Cat. No. 364979, Becton, Dickinson and Company, Franklin Lakes, NJ, USA) and allowed to clot at room temperature for different durations: 5 min for low-clotting samples, 15 min for moderate-clotting samples, and 20 min for high-clotting samples. All samples were gently mixed to ensure homogeneity before downstream CTC enrichment and analysis.

### 2.6. Serum CA 15-3, CA 125 and CEA Assays

Serum CA 15-3, CA 125, and CEA were performed according to the manufacturer’s instructions and using a cobas^®^ e 801 analyzer (Roche Diagnostics Ltd., Rotkreuz, Switzerland). The cut-off values were 43 U/mL for CA 15-3, 55 U/mL for CA 125, and 4.6 ng/mL for CEA.

### 2.7. Statistical Analysis

One-way ANOVA or unpaired Student’s *t*-test was performed for statistical analysis of cancer cell recovery rate and leukocyte depletion rate. Statistical analysis of the progression-free survival (PFS) and overall survival (OS) were determined by the log-rank test. PFS was calculated from the date of CTC testing to the date of the first evidence of disease progression (including local recurrence, distant metastasis, a significant increase in tumor markers or death). Patients who remained progression-free were censored at the date of the last follow-up. Analyses of the other data were performed by Mann-Whitney U test using GraphPad Prism 8 (GraphPad Software, Boston, MA, USA). The data represent the median ± interquartile range. A *p*-value < 0.05 was considered statistically significant.

## 3. Results

### 3.1. Reproducibility and Stability of the Chiline CATCH^®^ Circulating Target Cell Enrichment System

**Figure 2:**
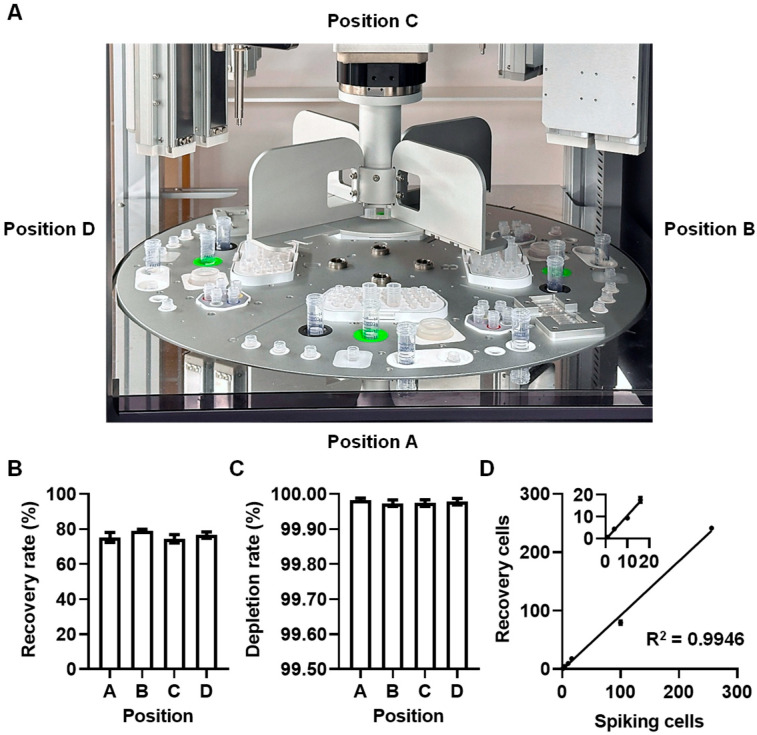
Reproducibility and stability of the Chiline CATCH^®^ Circulating Target Cell Enrichment System. (**A**) Schematic illustration of the four identical working units of the Chiline CATCH^®^ Circulating Target Cell Enrichment System. (**B**,**C**) Recovery rate and leukocyte depletion rate obtained from each working unit. Data are presented as mean ± SEM from 5 independent experiments. (**D**) SKBR3 cells pre-labeled with Calcein red-orange dye were spiked into NB4 cells at defined cell numbers (1, 4, 10, 16, 100, and 256 cells). Following CTC enrichment using the Chiline CATCH^®^ Circulating Target Cell Enrichment System, SKBR3 cells were identified by positive staining for calcein red-orange and Hoechst 33342. Linear regression analysis demonstrated excellent linearity between expected and detected SKBR3 cell numbers (R^2^ = 0.9946, *p* < 0.0001).

To reduce operator-dependent variability and ensure procedural consistency, the Chiline CATCH^®^ Circulating Target Cell Enrichment System was used in this study. This automated negative selection platform comprises four identical working units designed for CTC enrichment and enumeration (Figure 2A). To minimize inter-donor variability associated with whole blood samples and improve experimental reproducibility, NB4 cells were used as a standardized WBCs background in place of whole blood. Spike-in experiments were conducted in five independent runs using SKBR3 cells to evaluate CTC recovery and NB4 cells to assess leukocyte depletion, thereby determining the equivalence and consistency of the four working units.

The mean recovery rates for units A, B, C, and D were 75.3%, 79.1%, 74.5%, and 76.6%, respectively (Figure 2B). The overall mean recovery rate across all working units was 76.4%, with a coefficient of variation (CV) of 6.13%. The margin of equivalence, Δ, was set at 10%, and the predefined acceptable range for differences in recovery rate among the four working units was −10% to 10%. Using the Two One-Sided Tests (TOST) method, pairwise comparisons among the four working units yielded 90% confidence intervals (CIs) within the predefined equivalence margins (Table 1). These data indicate that there was no systematic performance difference among the working units and that the observed variability was acceptable for CTC enrichment assays.

The mean depletion rates for units A, B, C, and D were 99.983%, 99.974%, 99.974%, and 99.978%, respectively (Figure 2C). The mean depletion rate across all working units was 99.977%, with a CV of 0.02%, demonstrating highly consistent depletion of background cells in each working unit.

The linearity of SKBR3 cell recovery was evaluated across a dynamic range of spiked SKBR3 cell numbers in an NB4 cell background. The recovered SKBR3 cell counts in the NB4 background containing 1, 4, 10, 16, 100, and 256 SKBR3 cells were 1 ± 0.3, 4 ± 0.9, 9 ± 0.9, 18 ± 1.3, 79 ± 3.8, and 241 ± 1.7, respectively (Figure 2D). Linear regression analysis showed a strong fit (R^2^ = 0.9946, *p* < 0.0001), confirming a robust linear relationship between spiked and recovered SKBR3 cell counts. These data confirm the reproducibility and stability of the Chiline CATCH^®^ Circulating Target Cell Enrichment System.

Building on this linearity assessment, the sensitivity of the system was further evaluated to determine the LoD. SKBR3 cells were spiked at low numbers (1–5 cells) into NB4 background and each condition was tested in quadruplicate. Detection was only considered successful if at least 2 SKBR3 cells were recovered in a given replicate (Table 2). Based on these results, the LoD of the system was determined to be 2 SKBR3 cells per reaction, confirming that the system can reliably detect very low numbers of target cells.

### 3.2. CTC Enrichment Performance Is Unaffected by Hemolysis or Lipidemia but Impaired by Blood Clotting

**Figure 3:**
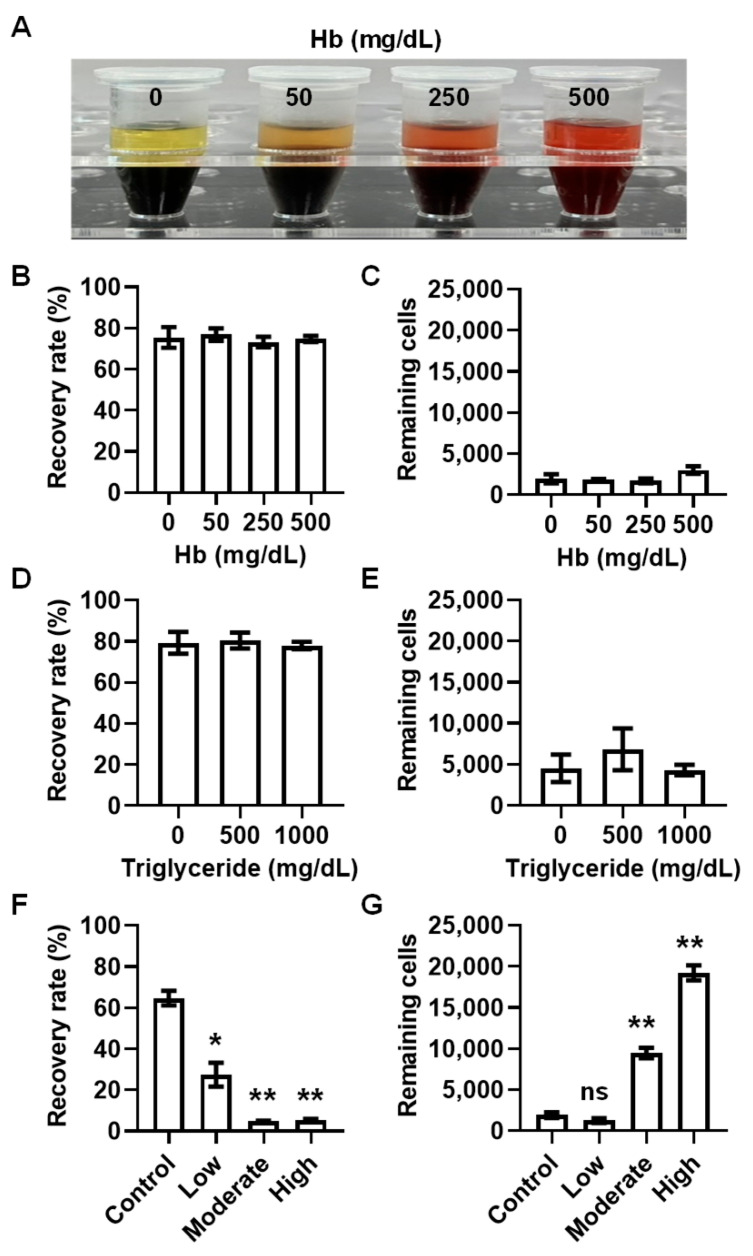
CTC enrichment performance is unaffected by hemolysis or lipidemia but impaired by blood clotting. (**A**) Schematic illustration of non-hemolyzed control blood samples and hemolyzed samples containing hemoglobin (Hb) at concentrations of 50, 250, and 500 mg/dL. (**B**,**C**) Recovery rate and remaining CD45-depleted cells in non-hemolyzed control samples and hemolyzed samples. Data are presented as mean ± SEM from 3 independent experiments. No significant differences were observed among groups (one-way ANOVA, *p* > 0.05). (**D**,**E**) Recovery rate and remaining CD45-depleted cells in samples supplemented with triglycerides at concentrations of 0, 500, and 1000 mg/dL, representing lipidemic conditions. Data are presented as mean ± SEM from 3 independent experiments. No significant differences were observed among groups (one-way ANOVA, *p* > 0.05). (**F**,**G**) Recovery rate and remaining CD45-depleted cells in clotting blood samples compared with non-clotted control samples. CTC enrichment and leukocyte depletion performance was significantly impaired in clotting samples (unpaired *t*-test; ns, no significance, * *p* < 0.05, ** *p* < 0.01). Data are presented as mean ± SEM from 2 independent experiments.

Hemolysis and lipidemia are common interfering factors in liquid biopsies and are also one of the reasons for specimen rejection [48,49]. To determine whether hemolyzed blood samples affected tumor cell recovery and leukocyte depletion performance, hemolyzed samples with hemoglobin concentrations of 50, 250, and 500 mg/dL were defined according to the Hemolysis Reference Palette, representing mild, moderate, and severe hemolysis, respectively (Figure 3A). The mean recovery rates for blood samples with hemoglobin concentrations of 0, 50, 250, and 500 mg/dL were 75.4%, 76.8%, 73.2%, and 74.7%, respectively (Figure 3B). No statistically significant differences were observed between recovery rates from hemolyzed samples and those from non-hemolyzed control samples at any tested hemolysis level (*p* > 0.05, one-way ANOVA), indicating that hemolysis did not appreciably affect assay recovery (Figure 3B). The normal adult WBC count ranges up to 11,000 cells/µL. The depletion rate exceeded 99.9% across all tested conditions (Figure 2C), resulting in a maximum of 22,000 remaining cells after enrichment, which was set as the acceptance criterion. The mean ± SEM remaining cell counts were 1947 ± 553, 1795 ± 135, 1703 ± 286, and 3012 ± 453 cells for 0, 50, 250, and 500 mg/dL hemoglobin groups, respectively. All hemolyzed and non-hemolyzed samples had remaining CD45-depleted cells below 22,000 cells (Figure 3C), and no statistically significant differences were observed among the different hemoglobin concentrations (*p* > 0.05, one-way ANOVA), indicating that hemolysis did not adversely affect leukocyte depletion performance.

To assess the impact of lipidemia, blood samples were supplemented with triglycerides at concentrations of 500 and 1000 mg/dL to simulate moderate and severe hypertriglyceridemia, respectively. The mean recovery rates were 79.2%, 80.4%, and 77.9% for 0, 500, and 1000 mg/dL triglyceride groups, respectively (Figure 3D). No statistically significant differences in recovery rates were observed among the groups (*p* > 0.05, one-way ANOVA), indicating that triglyceride supplementation did not adversely affect tumor cell recovery. The mean ± SEM remaining cell counts were 4523 ± 1666, 6821 ± 2532, and 4313 ± 623 cells for 0, 500, and 1000 mg/dL triglyceride groups, respectively (Figure 3E). All lipidemia samples had remaining CD45-depleted cells below 22,000 cells, and no statistically significant differences were observed among the different triglycerides concentrations (*p* > 0.05, one-way ANOVA) (Figure 3E). Collectively, these results demonstrate that the performance of the CTC enrichment system is not adversely affected by either hemolysis or lipidemia.

In contrast, the recovery rate was dramatically decreased in clotting samples, with mean recovery rates of 27.4%, 4.7%, and 5.1% in the low-, moderate-, and high-clotting groups, respectively, showing a statistically significant reduction compared with non-clotted samples (Figure 3F). For leukocyte depletion performance, the mean ± SEM remaining cell counts were 1910 ± 351, 1246 ± 292, 9455 ± 646, and 19,202 ± 905 cells for the control, low-, moderate-, and high-clotting groups, respectively (Figure 3G). Although the remaining cell counts in all groups were below 22,000, a clotting severity–dependent increase in remaining CD45-depleted cells was observed, indicating that blood clotting substantially impaired leukocyte depletion efficiency (Figure 3G). These data indicate that coagulated samples are not suitable for efficient CTC enrichment.

### 3.3. Basic Characteristics of Patients

**Figure 4:**
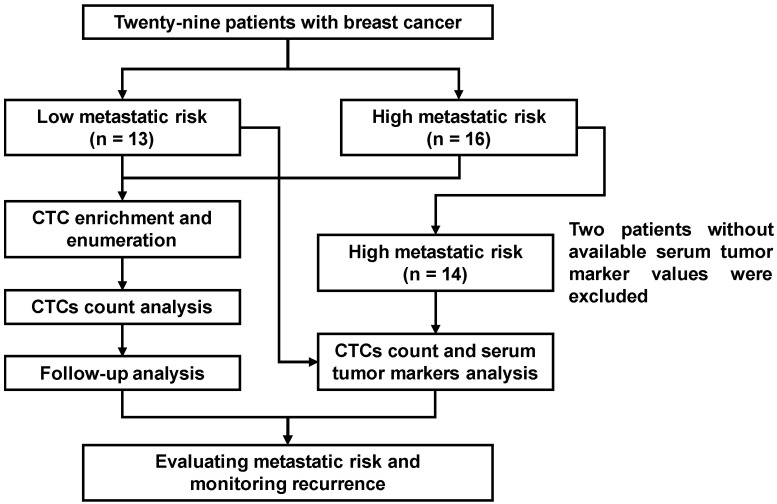
Flowchart of the study subjects, including exclusion criteria.

After validation of system performance under various sample conditions, the CTC enrichment platform was applied to clinical specimens. A total of 29 female patients with BC were enrolled to assess the diagnostic value of CTCs for evaluating metastatic risk and recurrence (Figure 4). The basic characteristics of patients enrolled in this study are shown in Table 3. The median age of patients was 52 years, with an interquartile range (IQR) of 49–61 years. The distribution of tumor-node-metastasis (TNM) staging was as follows: stage 0 (n = 1, 3.4%), stage I (n = 7, 24.1%), stage II (n = 5, 17.2%), stage III (n = 3, 10.3%), and stage IV (n = 13, 44.8%). Patients were stratified into two cohorts based on metastatic risk: a low-risk group (n = 13), comprising patients with stage 0-II disease or no recurrence for more than five years, and a high-risk group (n = 16), comprising patients with stage III-IV disease.

### 3.4. CSV^+^-CTCs Counts, but Not EpCAM^+^-CTCs Counts, Distinguish the Metastatic Risk of Patients with BC

**Figure 5:**
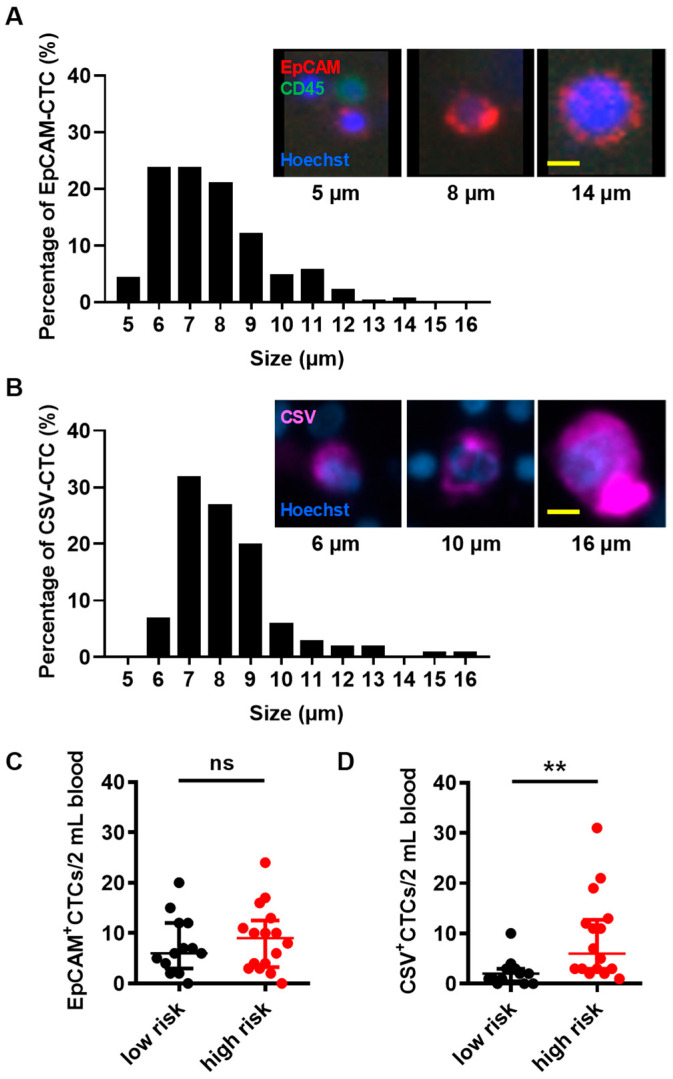
Size distributions of EpCAM^+^-CTCs and CSV^+^-CTCs and the scatter dot plots of EpCAM^+^-CTC and CSV^+^-CTC counts in the indicated patient groups. (**A**,**B**) Immunofluorescence staining was used to identify EpCAM^+^-CTCs and CSV^+^-CTCs with anti-EpCAM and anti-CSV antibodies. Cells positive for Hoechst 33342 staining were considered intact nucleated cells. Cells positive for both Hoechst 33342 and FITC-CD45 staining (green) were identified as intact leukocytes. Scale bar = 5 μm. The histograms show the percentage of EpCAM^+^-CTCs or CSV^+^-CTCs at each 1-μm diameter interval. (**C**,**D**) Scatter dot plots of EpCAM^+^-CTCs counts (panel **C**) and CSV^+^-CTCs counts (panel **D**) in patients with BC at low metastatic risk (n = 13) and high metastatic risk. Statistical analysis was performed by using the Mann-Whitney U test. Horizontal lines indicate the median ± interquartile range for each group. ns, no significance. ** *p* < 0.01.

The EMT is a process in which epithelial cells lose their cell polarity and cell-cell adhesion while acquiring migratory and invasive mesenchymal properties. EMT is associated with cancer progression and metastasis [50,51]. EpCAM is a marker of non-EMT cells, whereas CSV is a marker of EMT cells [31]. In this study, EpCAM and CSV were used to quantify non-EMT and EMT CTCs, respectively. Immunofluorescence staining was performed to identify EpCAM^+^-CTCs and CSV^+^-CTCs within the remaining CD45-depleted cell population. The diameters of EpCAM^+^-CTCs ranged from 5 to 14 μm, whereas those of CSV^+^-CTCs ranged from 6 to 16 μm, reflecting the size distributions of non-EMT and EMT CTCs, respectively (Figure 5A,B). Analysis of size distributions showed that EpCAM^+^-CTCs were predominantly 6–8 μm in diameter, whereas CSV^+^-CTCs were more broadly distributed, with most cells falling in the 7–9 μm range (Figure 5A,B). When comparing BC patients with low versus high metastatic risk, the counts of EpCAM^+^-CTCs and patient age were not significantly different (median age: 52 [IQR 50–57] vs. 53 [IQR 42–65] years; median EpCAM^+^-CTCs count: 6 [IQR 4–12] vs. 9 [IQR 4–12] cells/2 mL) (Figure 5C and Table 4). In contrast, CSV^+^-CTCs counts were significantly increased in the high metastatic risk group compared with the low metastatic risk group (median 6 [IQR 3–12] vs. 2 [IQR 1–3] cells/2 mL; *p* < 0.01) **(**Figure 5D and Table 4). These results indicate that CSV^+^-CTCs count, but not age or EpCAM^+^-CTCs count, can distinguish the metastatic risk of patients with BC.

**Figure 6:**
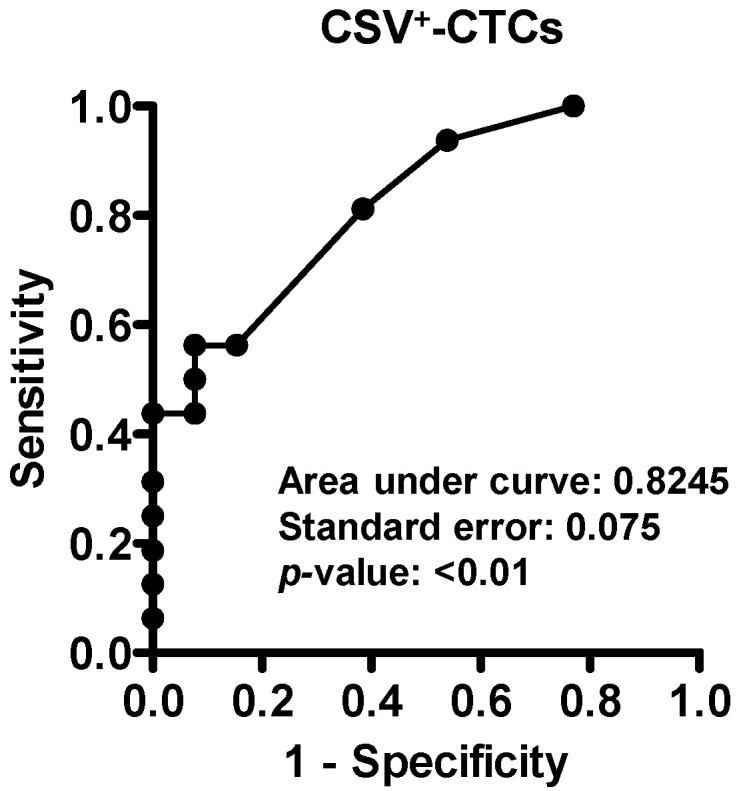
ROC analysis for discrimination between low- and high-metastatic-risk patients with BC based on CSV^+^-CTC counts.

A receiver operating characteristic (ROC) analysis was performed to determine the optimal CSV^+^-CTC cut-off for predicting metastatic risk in BC patients. The area under the curve (AUC) was 0.8245 (*p* < 0.01, Figure 6), indicating good diagnostic performance. Several candidate cut-off values are listed in Table 5. When the CSV^+^-CTC cut-off was set at >4.5 cells/2 mL of blood, the Youden’s index [52] was maximized, yielding a sensitivity of 0.56 and specificity of 0.92 (Table 5). These data suggest that BC patients with CSV^+^-CTC counts ≤ 4.5 cells/2 mL are considered at lower metastatic risk, whereas those with counts > 4.5 cells/2 mL are considered at higher metastatic risk.

### 3.5. Evaluation of CSV^+^-CTCs and Serum Tumor Markers in High Metastatic Risk BC Patients

CA 15–3, CA 125, and CEA are commonly used serum tumor markers for monitoring treatment response, disease recurrence, and metastasis in BC patients [13,14,15,16,17]. To further evaluate the clinical utility of CSV^+^-CTCs, their diagnostic performance was compared with that of these serum tumor markers. The cut-off values were set at 4.5 cells/2 mL blood for CSV^+^-CTCs, 43 U/mL for CA 15-3, 55 U/mL for CA 125, and 4.6 ng/mL for CEA. Two of the 16 high metastatic risk patients lacked complete serum tumor marker data and were therefore excluded from the analysis. Using a cut-off of >4.5 CSV^+^-CTCs/2 mL blood, the true positive rate for identifying high metastatic risk patients was 64.3%. In contrast, the true positive rates for CA 15-3, CA 125, and CEA were 28.6%, 28.6%, and 21.4%, respectively (Table 6). These results indicate that CSV^+^-CTCs have superior diagnostic performance to conventional serum tumor markers for distinguishing patients with BC at high metastatic risk.

Furthermore, when at least one parameter (CSV^+^-CTCs or any serum tumor marker) exceeded its respective cut-off value, the true positive rate increased to 85.7% among high metastatic risk BC patients (Table 6). These findings suggest that combining CSV^+^-CTCs with serum tumor markers improves detection capability compared with the use of individual markers alone for identifying BC patients at high risk of metastasis.

### 3.6. Representative Case of Early Recurrence Detected by CSV^+^-CTCs

Among low metastatic-risk BC patients, the CSV^+^-CTC count was below the cut-off value in 12 of 13 cases, yielding a true negative rate of 92.3%. In this group, all conventional serum tumor markers (CA 15-3, CA 125, and CEA) were below their respective cut-off values, corresponding to true negative rates of 100% (Table 6). Notably, one low metastatic-risk patient (ID: C1355) showed a positive CSV^+^-CTC result, with a count of 10 cells/2 mL of blood, exceeding the predefined cut-off value. This patient was therefore subjected to continuous clinical monitoring. Seven months later, a granulation was detected near the previously operated breast, and subsequent biopsy confirmed disease recurrence. The patient then underwent surgery followed by chemotherapy and dual-targeted therapy.

**Figure 7:**
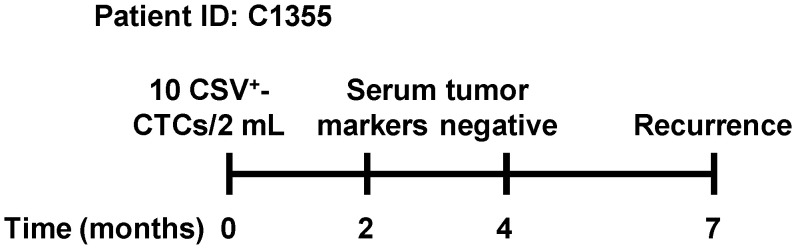
Timeline of clinical follow-up for patient ID: C1355.

Notably, serum tumor markers, including CA 15-3, CA 125, and CEA, were measured twice between the initial detection of CSV^+^-CTCs and confirmation of recurrence. All serum marker levels remained below their respective cut-off values throughout this period (Figure 7 and Table 7). This representative case highlights the potential clinical utility of CSV^+^-CTCs for early detection of BC recurrence, particularly when conventional serum tumor markers fail to indicate disease progression.

### 3.7. Kaplan-Meier Analysis of Progression-Free Survival (PFS) and Overall Survival (OS) in BC Patients Stratified by CSV^+^-CTC Count

**Figure 8:**
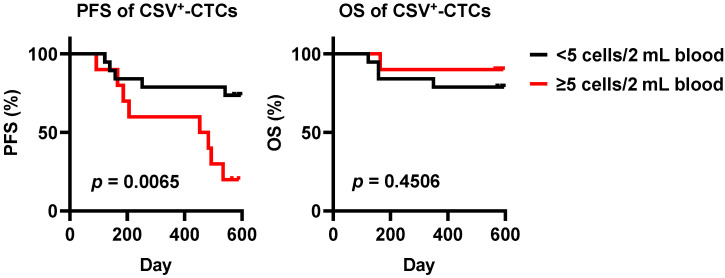
Kaplan–Meier analysis of progression-free survival (PFS) and overall survival (OS) in BC patients stratified by CSV^+^-CTC count. Patients were divided into two groups according to CSV^+^-CTC count: <5 cells/2 mL blood (black line) and ≥5 cells/2 mL blood (red line).

With a maximum follow-up of 595 days, patients were stratified into two groups based on CSV^+^-CTC count (<5 and ≥5 cells/2 mL blood). Kaplan–Meier analysis revealed a significant difference in PFS between the groups, with patients in the CSV^+^-CTC ≥ 5 group showing poorer PFS than those in the CSV^+^-CTC < 5 group (log-rank test, *p* = 0.0065). In contrast, OS did not differ significantly between the two groups (log-rank test, *p* = 0.4506), indicating that elevated CSV^+^-CTCs counts were associated with disease progression but may not affect short-term overall survival within the current follow-up period (Figure 8).

## 4. Discussion

In this study, the automated negative selection platform Chiline CATCH^®^ Circulating Target Cell Enrichment System demonstrated reproducible and stable performance for CTC enrichment and enumeration and was applied to evaluate metastatic risk and recurrence in patients with BC. Unlike EpCAM^+^-CTC counts, CSV^+^-CTC counts distinguished patients with low and high metastatic risk. Using a cut-off of >4.5 CSV^+^-CTCs/2 mL blood, CSV^+^-CTCs showed better diagnostic performance than serum tumor markers, including CA 15-3, CA 125, and CEA, for metastatic risk stratification and recurrence monitoring. In addition, an elevated CSV^+^-CTC count (≥5 cells/2 mL blood) was significantly associated with worse PFS. Together, these findings support the potential utility of this platform for tracking metastatic risk and recurrence in BC.

Liquid biopsy has emerged as a powerful tool in oncology because of its non-invasive nature, its potential for early detection, its capacity for real-time monitoring of tumor dynamics and treatment response, its ability to support comprehensive molecular profiling, and its potential to overcome limitations in tissue accessibility [53,54,55]. Among liquid biopsy analytes, CTCs are widely applied for cancer screening, treatment monitoring, and prognostic assessment, representing a major advance in personalized medicine [56]. However, the clinical application of CTCs remains challenging because of their marked heterogeneity in phenotype, marker expression, and physical properties [57].

The currently FDA-approved CTC detection system, CellSearch, relies on positive selection using epithelial markers such as EpCAM. This approach has inherent limitations, particularly in detecting mesenchymal or EMT-like CTCs, which often downregulate epithelial markers during cancer progression and metastasis [41,58]. In addition to marker-dependent approaches, size-based filtration methods have been widely applied for CTC enrichment, typically employing pore sizes of approximately 8 μm based on the assumption that CTCs are larger than leukocytes. However, size-based filtration systems rely primarily on cell size and deformability, which may lead to the loss of smaller or more deformable CTCs, particularly EMT-like CTCs that often exhibit increased plasticity [59,60]. In the present study, EpCAM^+^-CTCs and CSV^+^-CTCs exhibited overlapping but distinct size distributions, with diameters ranging from 5 to 14 μm and 6 to 16 μm, respectively (Figure 5A,B). Notably, a considerable proportion of both epithelial and EMT-like CTCs fell below the conventional 8 μm filtration threshold, highlighting the potential underestimation of CTC burden by size-based enrichment methods.

In contrast, the automated negative selection platform used in this study, the Chiline CATCH^®^ Circulating Target Cell Enrichment System, depletes CD45^+^ leukocytes without relying on tumor-specific capture markers or physical size constraints. This marker-agnostic and size-independent strategy enables the unbiased enrichment of heterogeneous CTC populations in a single workflow, including epithelial, mesenchymal, and intermediate EMT phenotypes, which can subsequently be identified using anti-EpCAM and anti-CSV antibodies. Consistent with previous reports [61,62,63] that detected both EpCAM^+^-CTCs and CSV^+^-CTCs in the blood of BC patients, our data confirm that these CTC subpopulations are also readily detectable using the Chiline CATCH^®^ system. Collectively, this approach provides a more comprehensive representation of CTC heterogeneity than conventional positive selection or size-based filtration systems.

No significant difference in EpCAM^+^-CTC counts was observed between the low metastatic risk and the high metastatic risk of BC patients. In contrast, CSV^+^-CTC counts were significantly higher in patients with high metastatic risk than in those with low metastatic risk (Figure 5C,D). These findings suggest that CSV^+^-CTC count, but not EpCAM^+^-CTC count, can distinguish the metastatic risk of patients with BC. This observation is consistent with the finding that CSV^+^-CTC counts have greater sensitivity and specificity than EpCAM^+^-CTC counts for evaluating disease progression in patients with prostate cancer [41].

Focusing exclusively on CSV may underestimate the total mesenchymal CTC population if some cells express only intracellular Vimentin without surface translocation. However, the CSV-based identification strategy provides an important technical advantage in the present study. A recurring challenge in CTC isolation is the contamination of sample populations by leukocytes. Because leukocytes endogenously express high levels of cytoplasmic Vimentin, conventional intracellular staining can generate substantial background noise and requires complex exclusion strategies, such as CD45-negative gating. In contrast, CSV is expressed on the surface of cancer cells but is absent from the surface of normal blood cells [46]. This enables the monoclonal antibody clone 84-1 to identify CSV^+^-CTCs more specifically while reducing false-positive signals from residual leukocytes, particularly CD45^dim^ leukocytes, that may remain after negative selection. Detection of EMT-like CTCs is important for understanding cancer progression. Although intracellular Vimentin is a well-established mesenchymal marker, its presence alone does not fully capture metastatic potential. Partial translocation of Vimentin from the cytoplasm to the cell surface is not merely a structural change but may indicate a more aggressive phenotype with enhanced capacity to survive circulatory shear stress and facilitate distant organ colonization [39,46,64]. For this reason, even though a CSV-based strategy may underestimate the total mesenchymal CTC population, our study still indicates that CSV^+^-CTC counts are associated with metastatic risk stratification and recurrence in patients with BC.

Although CA 15-3, CA 125, and CEA are among the most commonly used biomarkers in BC [17,65], their limited sensitivity restricts their accuracy for detecting recurrence. Accordingly, the diagnosis of BC recurrence is better supported by combined measurement of serum tumor markers, such as CA 125, CEA, and cancer antigen 27-29 (CA 27-29), than by any single marker alone [65,66,67,68]. In addition, the American Society of Clinical Oncology also does not recommend tumor markers such as CA 15-3 or CA 27-29 for screening, diagnosis, staging, or routine monitoring for recurrence in BC [69]. In the present study, CSV^+^-CTCs (cut-off > 4.5 cells/2 mL blood) showed the highest true positive rate (64.3%) compared with CA 15-3 (28.6%), CA 125 (28.6%) and CEA (21.4%) in high metastatic risk BC patients. By combining the analysis of CSV^+^-CTCs and serum tumor markers, the true positive rate increased to 85.7%. When CSV^+^-CTCs were combined with serum tumor markers, the true-positive rate increased to 85.7%. Similar improvements in diagnostic performance have been reported when CTC analysis is combined with serum tumor markers [70,71]. For example, combining CTCs with CEA enhances clinical prediction in patients with non-small cell lung cancer [71].

In this study, one low metastatic-risk patient with BC (ID: C1355) showed an elevated CSV^+^-CTC count and developed disease recurrence seven months later, despite normal levels of conventional serum tumor markers (CA 15-3, CA 125, and CEA) (Figure 7 and Table 7). Stratification of all patients by CSV^+^-CTC count (<5 vs. ≥5 cells/2 mL blood) revealed a significant difference in PFS, with patients in the ≥5 group showing poorer PFS. In contrast, OS did not differ significantly between the two groups (Figure 8). The lack of a significant OS difference is likely attributable to the relatively short follow-up period (maximum 595 days) and the limited number of death events, which may have precluded the detection of OS differences, whereas PFS reflects earlier indicators of disease progression.

These findings are consistent with previous studies showing that elevated CTC counts, including EMT-like subpopulations, are associated with shorter PFS and OS in BC [72,73]. For example, in metastatic BC, baseline CTC counts ≥ 5 cells per 7.5 mL blood were associated with significantly poorer PFS and OS [72]. Moreover, meta-analyses indicate that EMT-phenotype CTCs are linked to adverse outcomes, underscoring the importance of capturing heterogeneous CTC populations for prognostic evaluation [74]. Taken together, our results suggest that elevated CSV^+^-CTCs are associated with disease progression and may enable earlier detection of recurrence than conventional serum tumor markers, even when short-term OS differences are not apparent. Larger studies with longer follow-up are warranted to validate the prognostic value of CSV^+^-CTCs for both PFS and OS in patients with BC.

## 5. Conclusions

This study demonstrates that an automated platform can enrich both epithelial CTCs (EpCAM^+^-CTCs) and mesenchymal CTCs (CSV^+^-CTCs) from peripheral blood. Compared with EpCAM^+^-CTC count, CSV^+^-CTC count was more closely associated with metastatic risk in patients with BC. Overall, CSV^+^-CTCs showed better diagnostic performance than serum tumor markers, including CA 15-3, CA 125, and CEA, for metastatic risk stratification and recurrence monitoring in this cohort. In addition, an elevated CSV^+^-CTC count (≥5 cells/2 mL blood) was significantly associated with worse PFS. These findings suggest that CSV^+^-CTCs may serve as a useful biomarker for metastatic risk stratification and recurrence monitoring in BC when measured using an automated negative selection platform.

## Figures and Tables

**Table 1:** Equivalence margins for the four working units.

Pair	Mean Difference	SE	90% CI Low ^a^	90% CI High ^a^
A vs. B	−3.8	2.99	−8.71	1.11
A vs. C	0.8	3.74	−5.36	6.96
A vs. D	−1.3	3.35	−6.81	4.21
B vs. C	4.6	2.56	0.39	8.81
B vs. D	2.5	1.94	−0.69	5.69
C vs. D	−2.1	2.98	−7.00	2.80

^a^ The margin of equivalence, Δ, was 10% and the range −10% to 10% was predefined as an acceptable range. SE, standard error.

**Table 2:** Number of SKBR3 cells recovered in the spiking test.

Spiking Test	1	2	3	4
No. of Cell Spiked	No. of Cell Recovery			
1	0	1	1	1
2	2	1	2	2
3	2	3	1	2
4	2	3	2	4
5	5	5	3	4

**Table 3:** Basic information of the patients enrolled in this study.

Variables	Median Years or No. of Patient	Interquartile Range or Percentage	Metastatic Risk
Age	52	49–61	
Gender, male/female	0/29		
Tumor-node-metastasis staging			
Stage 0	1	3.4	low
Stage I	7	24.1	low
Stage II	5	17.2	low
Stage III	3	10.3	high
Stage IV	13	44.8	high

**Table 4:** Basic characteristics and CTCs count of the study subjects.

Characteristics	Age (Year) ^a^	EpCAM^+^-CTCs/2 mL ^a^	CSV^+^-CTCs/2 mL ^a^
Patients (n = 29)			
Low metastatic risk (n = 13)	52 (50–57)	6 (4–12)	2 (1–3)
High metastatic risk (n = 16)	53 (42–65)	9 (4–12)	6 (3–12)
*p*-value ^b^	0.6137	0.6604	0.0030

^a^ Data represent the median and interquartile range for the indicated parameters. ^b^ Statistical analysis compared the low metastatic risk and the high metastatic risk group of BC patients.

**Table 5:** Youden’s index for different CSV^+^-CTC cut-off values.

CSV^+^-CTCs Cut-Off (per 2 mL)	Sensitivity	Specificity	Youden’s Index ^a^
>0.5	1.00	0.23	0.23
>1.5	0.94	0.46	0.40
>2.5	0.81	0.62	0.43
>3.5	0.56	0.85	0.41
>4.5	0.56	0.92	0.49
>6.0	0.50	0.92	0.42
>8.5	0.44	0.92	0.36
>10.5	0.44	1.00	0.44

^a^ Youden index = sensitivity + specificity − 1.

**Table 6:** Comparison of CTC counts and serum tumor markers for evaluating patients with BC.

Parameters	CSV^+^-CTCs (Cells/2 mL Blood)	CA 15-3 (U/mL)	CA 125 (U/mL)	CEA (ng/mL)	All
Cut-off value	>4.5	≥43	≥55	≥4.6	Dependence on parameters
Patients ^a^	Number of cases with above cut-off value (True positive rate)				
High metastatic risk (n = 14)	9 (64.3%)	4 (28.6%)	4 (28.6%)	3 (21.4%)	12 ^b^ (85.7%)
	Number of cases with below cut-off value (True negative rate)				
Low metastatic risk (n = 13)	12 (92.3%)	13 (100%)	13 (100%)	13 (100%)	ND

^a^ Only patients with complete data were included in this table. ^b^ One of the parameters ≥ cut-off value in the high metastatic risk BC patients was included in the calculation. ND, not determined.

**Table 7:** Follow-up values of serum tumor markers in patient ID: C1355.

Patient ID	C1355	
Time (months) ^a^	2	4
Tumor markers		
CA 15-3 (U/mL)	15.0	13.1
CA 125 (U/mL)	6.3	6.3
CEA (ng/mL)	0.9	0.5

^a^ Day 1 was defined as the date of the CSV^+^-CTC testing.

## Data Availability

Data are available from the corresponding author upon reasonable request.

## Supplementary Materials

The following supporting information can be downloaded at: https://www.mdpi.com/article/10.3390/diseases14040130/s1, Supplementary File S1: Detailed patient characteristics; Supplementary Figure S1: Specificity validation of the anti-EpCAM antibody.
