# Supplementary material for: Diagnostic and Prognostic Utility of Cell-Surface Vimentin Positive Circulating Tumor Cells in Breast Cancer Using an Automated Negative Selection Platform
Source: Diseases. 2026 Apr 3;14(4):130. doi: 10.3390/diseases14040130 (PMC13115473; doi:10.3390/diseases14040130)
Supplement: Supplementary file 1 [file diseases-14-00130-s001.zip › Supplementary Figure S1-20260403.pdf]

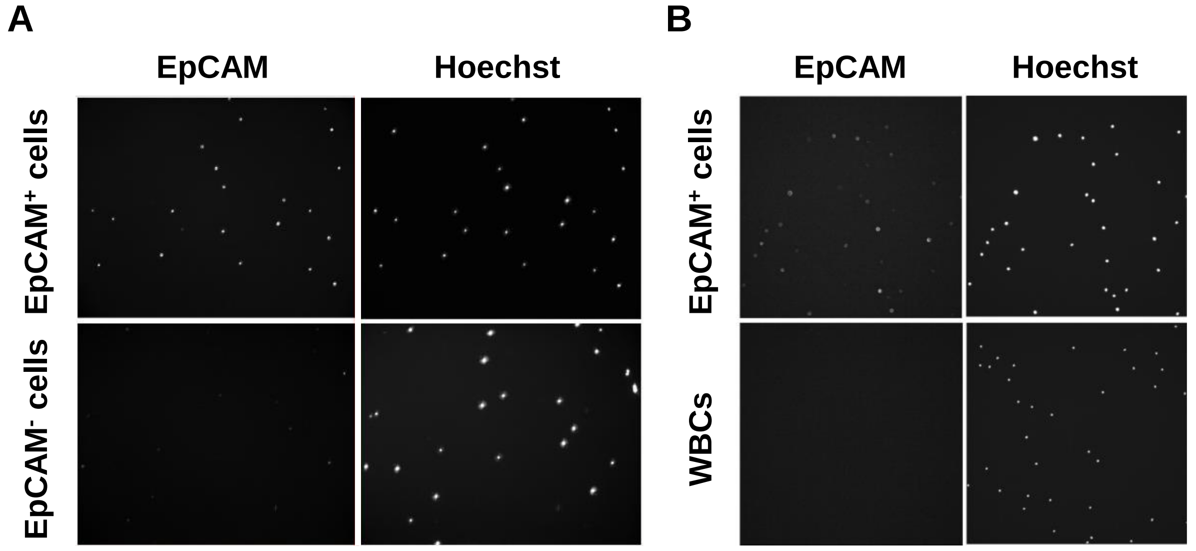

Supplementary Figure S1. Specificity validation of the anti-EpCAM antibody. (A-B) The specificity of the anti-EpCAM antibody was evaluated using EpCAM^{+} cells, CRISPR-mediated EpCAM-knockout (EpCAM^{-}) cells, and primary white blood cells (WBCs). These data indicate that the formulated antibody is highly specific for EpCAM, as it showed no non-specific signal in the knockout control and no cross-reactivity with WBCs.

Yeh *et al*. Supplementary Figure S1
